# Making change happen in criminal justice settings: leveraging implementation science to improve mental health care

**DOI:** 10.1186/s40352-020-00122-6

**Published:** 2020-09-06

**Authors:** Melissa J. Zielinski, M. Kathryn Allison, Lauren Brinkley-Rubinstein, Geoffrey Curran, Nickolas D. Zaller, Jo Ann E. Kirchner

**Affiliations:** 1 Psychiatric Research Institute, University of Arkansas for Medical Sciences, 4301 W. Markham Street, Little Rock, AR 72205 USA; 2 University of Arkansas, Fayetteville, AR USA; 3 University of North Carolina, Chapel Hill, NC USA; 4 Central Arkansas Veterans Healthcare System, North Little Rock, AR USA

**Keywords:** Implementation science, Criminal justice, Prison, Jail, Mental health, Substance Use

## Abstract

**Background:**

It is a constitutional right to receive health care, including mental health care, while incarcerated. Yet, even basic evidence-based mental health care practices have not been routinely integrated into criminal justice (CJ) settings. Strategies from implementation science, or the study of methods for integrating evidence-based practices into routine care, can accelerate uptake of established interventions within low-resource, high-need settings such as prisons and jails. However, most studies of mental health practices in CJ settings do not use implementation frameworks to guide efforts to integrate treatments, systematically select or report implementation strategies, or evaluate the effectiveness of strategies used.

**Case presentations:**

After introducing implementation science and articulating the rationale for its application within CJ settings, we provide two illustrative case examples of efforts to integrate mental health interventions within CJ settings. Each case example demonstrates how an implementation framework either was applied or could have been applied to promote intervention adoption. The first focuses on poor implementation of a mental health screener in a county jail, retrospectively highlighting how use of a determinants framework (e.g., the Consolidated Framework for Implementation Research [CFIR]) could help staff identify factors that led to the implementation failure. The second describes an investigator-initiated research study that used a process framework (the Exploration, Preparation, Implementation, Sustainment [EPIS] framework) to systematically investigate and document the factors that led to successful implementation of a psychotherapy group for survivors of sexual violence in a women’s community corrections center. Both are presented in accessible language, as our goal is that this article can be used as a primer for justice health researchers, community partners, and correctional leadership who are unfamiliar with implementation science.

**Conclusions:**

Scientific research on the application of implementation science to justice settings is growing, but lags behind the work done in health systems. Given the tremendous need for mental and behavioral health intervention across the full spectrum of justice settings, information on how to successfully implement evidence-based intervention and prevention efforts is sorely needed but possible to obtain with greater integration of knowledge from implementation science.

## Background

Over half of people who are incarcerated meet criteria for at least one mental illness (Fazel & Danesh, 2002; Fazel, Yoon, & Hayes, 2017). Substance use disorders and other mental illnesses that are associated with trauma (e.g., posttraumatic stress disorder, major depressive disorder, personality disorders) are particularly common (Fazel & Danesh, 2002; Fazel et al., 2017; Karlsson & Zielinski, 2020). Beyond the obvious personal health implications of mental illness, it can also contribute directly to the outcomes that incarceration is supposed to deter. For example, studies have found that non-substance-related mental disorders—both alone and in combination with substance use disorders—are associated with arrests for new crimes (Houser, Saum, & Hiller, 2019; Sadeh & McNiel, 2015). Posttraumatic stress disorder may be particularly strongly related to crime and incarceration (Proctor, Alvarez de la Campa, Medina-Reyes, & Hoffmann, 2017; Sadeh & McNiel, 2015), while other disorders have a less pronounced relationship (Wilson & Wood, 2014). Mental illness also increases the many challenges people face when returning to the community from incarceration (e.g., paying costly fees/fines, finding and maintaining employment, attending parole appointments).

However, even basic evidence-based practices relevant to tackling mental illness such as integrated screening based on validated instruments, are absent or inconsistently applied in many U.S. correctional facilities (Prison Health Care: Costs and Quality, 2017). Complex interventions, such as evidence-based psychotherapies, have had even less reach. The time is ripe for studies that aim to identify effective strategies for ensuring that criminal justice (CJ) settings implement evidence-based mental health practices. Improving carceral mental health care will in turn improve public health and decrease the barriers that people face when trying to rebuild their life post-incarceration.

The purpose of this paper is to provide basic information about *implementation science,* an emerging, interdisciplinary field dedicated to studying how research findings or clinical interventions are best adopted and integrated into routine practice (Eccles & Mittman, 2006). Because the paper is intended to be introductory and for readers whose knowledge of implementation science is limited, we present only a small portion of what implementation has to offer CJ settings. Citations and resources available for further learning appear at the end of the manuscript. We believe that increasing the knowledge of this field among CJ scholars and practitioners who may be in a position to advance the scientific knowledge on implementation strategies that are and are not effective in CJ settings is paramount to improving health care in our nation’s prisons and jails and increasing individuals’ odds of success during community re-entry. After providing a basic introduction to select foundational implementation science terms, we provide two illustrative case examples that demonstrate practical application. These case examples focus on: (1) implementing a screening measure in a large county jail and (2) identifying factors that have contributed to successful implementation and maintenance of a group trauma therapy in a community corrections center for women via a retrospective process evaluation.

### Introduction to implementation science

*Implementation science* is the scientific study of methods for integrating evidence-based practices into routine care (Eccles & Mittman, 2006). The field arose in response to growing awareness that many evidence-based interventions either never make it into routine practice or take nearly two decades to do so (Balas & Boren, 2000; Morris, Wooding, & Grant, 2011). The field also responded to growing evidence that interventions developed and tested in tightly-control efficacy trials often require some level of customization to be successfully integrated into and sustained in their intended care setting by providing methods for assessing and documenting intervention adaptation (Chambers & Norton, 2016; Stirman, Baumann, & Miller, 2019).

Implementation science has been used broadly in fields ranging from agriculture to education to healthcare (Institute of Medicine, 2001). Here, we will focus our efforts on applying implementation science to mental health care innovations (typically called “interventions” outside of implementation science) that there has been at least some push to implement within CJ settings (e.g., population-level screenings, evidence-based therapies) Harner, Budescu, Gillihan, Riley, & Foa, 2015; Rich et al., 2015; Taxman, Cropsey, Young, & Wexler, 2007). Proctor and colleagues (2009, p. 24) noted that one of the most critical issues in mental health services research is “the gap between what is known about effective treatment and what is provided to and experienced by consumers in routine care in community practice settings”—a gap that is notably wider in CJ settings in the U.S. (Abramsky & Fellner, 2003). While correctional facilities in the U.S. have been considered the largest providers of mental health services in the country (Torrey, 1995), access to and quality of these services in prisons and jails are inconsistent and/or inadequate due to known implementation barriers, like limited resources, staff, and funding as well as features of the broader sociopolitical context (Adams & Ferrandino, 2008; Gonzalez & Connell, 2014; Scott-Hayward, 2009; Young, Farrell, Henderson, & Taxman, 2009). Implementation science offers an opportunity to improve mental and behavioral health outcomes among incarcerated populations by identifying the factors that influence the uptake and sustained use of evidence-based mental health services and matching strategies to implementation influences. Implementation science can also help CJ settings evaluate the success of implementation strategies that aim to increase access to quality mental health care by providing guidance about appropriate outcomes to examine (e.g., Proctor et al., 2011). This paper serves to define implementation science terminology and demonstrate its application to the field of mental health treatment in CJ settings. Important terms are defined in Table 1 and reviewed in paragraph form below to facilitate greater understanding of the relations between terms.

**Table 1:** Introduction to implementation science terms

*Term*	*Refers to*	*Example(s)*
Implementation science	The scientific study of methods for integrating evidence-based practices into routine care.	–
Implementation strategies	Approaches used to assist with integration of the innovation.	• Educational outreach visits • Audit and feedback • Revision of professional roles
Implementation tools	Products that assist with the implementation of the intervention	• Comprehensive toolkits • Decision-trees for choosing amongst several treatments
Intervention	*Selected* implementation strategies and/or tools that may be used independently or in combination to support integration of evidence-based practices.	• Staff training • Providing clinical supervision • Providing technical assistance
Innovation	The evidence-based practices being implemented.	• Mental health screening • Cognitive Behavioral Therapy
Formative evaluation	Systematic assessments used to obtain knowledge of factors that may affect implementation.	• Stakeholder interviews or focus groups • Needs assessment survey • Observation
Factors	Barriers and facilitators (i.e., determinants of practice)	• Intervention cost • Available resources • Leadership engagement • Individual knowledge and beliefs about the intervention

#### Terminology

Rather than a single event, implementation should be thought of as a set of activities with the goal of putting something into practice (Fixsen, Naoom, Blasé, Friedman, & Wallace, 2005). Foundational to implementation science are implementation *strategies* and *tools*, which aim to support the uptake of *innovations* (i.e., the evidence-based practices being implemented). *Implementation strategies* can be selected based on knowledge gained from *formative evaluations* (e.g., assessments of current practices, anticipated implementation barriers/facilitators, and current stakeholder priorities), that often precede implementation efforts, but that may also be used during the implementation process (Stetler et al., 2006). Strategies can also be selected based on *conceptual models, theories,* and *frameworks*, as described below.

*Implementation strategies* are used to support intervention uptake at target sites and are evaluated on provider- and systems-level behaviors (e.g., innovation adoption, sustainment, and fidelity) and perceptions (e.g. perceived feasibility and acceptability). Example strategies include providing educational sessions about and/or technical assistance in the clinical innovation, altering incentive structures to reward use of the clinical innovation, using train-the-trainer strategies to increase capacity, and conducting performance audits and providing feedback (Powell et al., 2015). *Implementation tools* are specific products that assist with implementation of the innovation; examples could range from simple informational handouts to fully elaborated toolkits, which provide software programs that assist with automating the innovation.

Implementation science also involves the application of theory-driven *conceptual models* and *frameworks*. A recent systematic review identified 49 different implementation frameworks for a healthcare innovation (Moullin, Sabater-Hernández, Fernandez-Llimos, & Benrimoj, 2015). Implementation models and frameworks can be selected based on the innovation (interventions, guidelines, knowledge, evidence-based practice model, and implementation programs) or stage of implementation, such as pre-implementation (development, communication, and exploration) or active implementation (installation, operation, and sustainability) (Moullin et al., 2015). Recognizing both the type of innovation and the stage of implementation can aid in selecting the most appropriate theory, model, or framework. Nilsen (2015) describes three overarching aims of theoretical approaches in implementation science:

1. plan, describe, or guide the implementation process [*process frameworks*];
2. identify factors that influence implementation outcomes or design implementation interventions [*determinants frameworks*, *classic theories*, and *implementation theories*]; and,
3. evaluate or measure the success of implementation [*evaluation frameworks*].

### Research on implementation strategies and outcomes in criminal justice settings

Research that has applied implementation science in CJ settings has primarily measured implementation-related outcomes (e.g., the number of people who have received an intervention, innovation fidelity) or used formative evaluations to adapt interventions and/or identify anticipated barriers and facilitators to innovation implementation. For example, a study of an HIV/STI prevention intervention for incarcerated women by Fogel et al. (2015) was based on formative research in which interviews and focus groups with correctional stakeholders were used to guide adaptation of a pre-existing curriculum specifically for the prison setting (Fasula et al., 2013). Studies of interpersonal psychotherapy for depression in the prison setting conducted by Johnson et al. (2016, 2019) have integrated assessments of implementation factors such as treatment acceptability, fidelity, and cost effectiveness, as well as barriers/facilitators to implementation (Johnson et al., 2016, 2019). A recent study by Kim and colleagues (Kim et al., 2019) detailed a novel application of process mapping to examine implementation of peer support for Veterans leaving incarceration.

These studies and others are evidence that implementation outcomes and determinants have received some attention in CJ research. Assessment of barriers and facilitators to implementation of various evidence-based treatments in CJ settings have been especially common. However, the potential for implementation science models, strategies, and measures to aid CJ researchers and practitioners in more quickly promoting uptake of evidence-based innovations that have potential to improve outcomes of justice-involved clients has been largely untapped. For example, implementation science research has identified a portfolio of strategies that have potential to enhance the uptake and sustainment of effective practices in real-world settings (Powell et al., 2015), including low-resource settings (Kirchner et al., 2014). However, studies of whether the implementation models, strategies, and measures developed for other contexts generalize to CJ settings are lacking. This is an important literature gap given that the factors that influence adoption of evidence-based practices may differ by innovation and thus require use of relevant strategies (Ducharme, Knudsen, Roman, & Johnson, 2007).

The National Institute of Drug Abuse (NIDA)-funded Criminal Justice Drug Abuse Treatment Studies (CJ-DATS) and Juvenile Justice-Translational Research on Interventions for Adolescents in the Legal System (JJ-TRIALS) study are two notable examples of implementation science research in CJ settings. CJ-DATS focused on implementation of drug use treatment within adult CJ settings (Ducharme, Chandler, & Wiley, 2013). The novel implementation strategies that were examined in CJ-DATS included but were not limited to organizational linkage interventions for facilitating referrals to medication assisted treatment (Friedmann et al., 2013, 2015) and external coaching through a local change team to improve prevention, detection, and treatment for HIV in prisons (Belenko et al., 2013); the experimental strategies in both of the aforementioned studies were compared to standalone staff training as a control condition. Some studies within CJ-DATS also examined the implementation process; for example, work by Taxman and colleagues examined how five justice settings implemented contingency management, an evidence-based intervention for initiating drug abstinence that is rarely used in practice, following training in the intervention (Rudes et al., 2012; Taxman & Rudes, 2013). Research done within JJ-TRIALS compared two novel implementation interventions (one comprised of several evidenced-based strategies described as *Core* and an *Enhanced* condition comprised of all core strategies plus active facilitation) for improving evidence-based screening, assessment, and linkage to drug treatment in juvenile justice settings (Knight et al., 2015). This JJ-TRIALS study was also unique in that it used an implementation science framework from project inception through completion—including to select implementation strategies, define research questions, and select measurement tools (Becan et al., 2018). NIDA has also recently funded a third initiative, the Justice Community Opioid Innovation Network (JCOIN), which aims to improve access to high-quality care for opioid use disorder in CJ settings and also integrates implementation-related research questions to improve knowledge on how to implement such interventions elsewhere in the future.

These large, federally-funded trials are only one example of how implementation science can advance knowledge that is relevant to CJ researchers and practitioners. Routine integration of implementation science principles, models, and strategies in health-oriented research in CJ settings would exponentially increase knowledge on how to speed the translation and adoption of evidence-based behavioral health practices in this context.

## Case presentation

Based on work of the co-authors, we present two illustrative case examples of efforts to integrate behavioral health interventions within CJ settings. Each case example demonstrates how an implementation framework either was applied or could have been applied to promote adoption of mental health innovations to the target setting. The first case example explains how a *determinants framework* could help county jail staff identify factors that may have influenced poor implementation of a behavioral health screener that they attempted to use. The second case example describes an investigator-initiated research study that was designed using a *process framework*, as the goal was to describe the implementation process that supported successful implementation of a psychotherapy group in a community corrections setting (see Table 2).

**Table 2:** Case examples

	*Case example*	*Setting*	*Framework*
Case example 1	Screening for mental and behavioral health disorders	Jail	CFIR (determinants framework)
Case example 2	Process evaluation of a group trauma therapy	Community Correction Center	EPIS (process framework)

### Case example 1 – failed implementation of screening for mental health disorders in a jail

#### Background

Before designing interventions, systems must have a way of identifying individuals who are in need of services. As previously described, studies of mental health among CJ-involved persons have found great need for behavioral health interventions such as cognitive behavioral therapies (Pearson, Lipton, Cleland, & Yee, 2002; Wilson, Draine, Hadley, Metraux, & Evans, 2011) and medication-assisted treatment (Brinkley-Rubinstein et al., 2018; Green et al., 2018) and prompted calls for integrated health screening (Rich, Allen, & Williams, 2015). However, efforts to integrate even relatively simple components to evidence-based care, such as screening, often fail without using strategies that overcome barriers to implementation.

#### Initial case example facts

Directed by county leadership, staff at a large county jail were charged with using a mental health screener to identify people in need of mental health services. Jail leadership wrote a policy memo directing staff to administer the screener to all people booked in to the jail and enter the results into the online booking system. When screener data was pulled from the online booking system several months later, it became clear that the screener had not been routinely implemented; leadership could not understand the lack of action on the part of the jail staff and struggled with what to do to improve screening rates.

#### Approach to case review

If facility leadership were familiar with implementation science, they would be more readily able to identify factors that may have influenced poor implementation of the screener by using a framework to seek more information about potential influences. Here, we apply the Consolidated Framework for Implementation Research (CFIR; Damschroder & Hagedorn, 2011) considering details that are known to us about this case (but which are not drawn from a formal research study) to illustrate how the framework could be used by leadership and/or an academic partner to gather knowledge and create a revised implementation plan. Of note, this case example is drawn from a retrospective appraisal of a naturally occurring implementation failure discussed during consultation with academic partners. All case details were drawn from self-reports by jail and county-level leaders. Greater available resources and/or time could facilitate even more depth of exploration—for example, through semi-structured interviews, administration of survey self-report measures to index CFIR constructs, and/or review of administrative data.

#### Examination of implementation failure using an implementation science lens

CFIR includes five domains: 1) the intervention characteristics, 2) the characteristics of individuals involved with the implementation process, 3) the inner setting, 4) the outer setting, and 5) the implementation process itself. By considering each domain, we narrow in on information that is relevant to failed implementation and that can be used to recommend strategies to address barriers and maximize factors that could optimize implementation. First, the screener was a 10-item screening tool (*intervention characteristics*) for which staff had received no training to administer. Leadership had reportedly not considered that training would be needed. Individual discussions with key stakeholders revealed that they did not perceive value in the screener and actually saw its use as a disadvantage because it increased intake time (*characteristics of individuals involved with the implementation process*). Also, though leadership had directed use of this screener, this was not adequately communicated during staff and/or shift meetings in the way that other high-priority changes in the facility were typically discussed. Therefore, staff perceived that leadership support for the change was low (*inner setting*). Additionally, several senior staff members expressed opposition to both use of the screener and to having to spend time aggregating reports (*characteristics of individuals involved with the implementation process*). This opposition was reportedly expressed verbally during staff meetings as well as passively through repeated non-response to requests for data by county-level leadership. The facility was highly understaffed (*inner setting*) and state funding for increasing care based on the results of the screener was not expected (*outer setting*) per the facility’s health administrator.

#### Revised implementation strategies

To address the knowledge gained through application of CFIR above, leadership could make several changes to their implementation plan to better match their approach to implementation barriers experienced. First, they might consider assigning screener administration to specific people who are already used to using assessment tools. For example, medical staff in the intake unit might be identified as the primary/appropriate people to administer the screener. Education about the screener and about the rationale for wanting to identify people with mental illness in the jail could be provided to improve perceptions of the screener as valuable; this same strategy may also be a method to implicitly or explicitly demonstrate leadership’s commitment to the initiative and to show that the screener can, in reality, be completed in less than 2 min in most cases. Leadership could also work with the vendor for their online booking system to automate the process of generating reports of screener results to reduce the burden this task on the already taxed senior staff expressing opposition. Screening rates could be re-assessed each month after these new strategies are being used to determine the effectiveness of the new implementation plan and consider whether there is a need to further refine the strategies used to support screener uptake.

Although the proposed solutions are hypothetical and we cannot report data on outcomes, this scenario illustrates how use of an implementation science framework can guide correctional staff in considering the many layers of influence on whether implementation efforts of various initiatives succeed or fail. The process of examining implementation failures in this way could be applied to any initiative being pursued in these settings—including the many mental and behavioral health interventions that address the needs of justice involved populations. Clearly, the approach outlined is a departure from traditional top-down implementation in which mandates may be delivered without designing and communicating an implementation plan that is likely to be successful in the setting; doing this by using an implementation science framework to guide decisions *prior* to launching the screener, in this example, would be an even greater improvement on the post-failure process we have described.

### Case example 2 – process evaluation of a group trauma therapy in a community corrections center

#### Background and initial case example facts

Through collaborations between a university psychology program, a domestic violence shelter, and a community corrections center for women, it was recognized that many incarcerated women have previously experienced sexual violence and may benefit from trauma therapy. In response to this identified need, a team from the university psychology program developed, provided, and evaluated a group therapy (*Survivors Healing from Abuse: Recovery through Exposure* [SHARE]) for incarcerated women who are survivors of sexual violence in the community corrections center, with the ultimate goal of improving access to trauma therapies in CJ settings. Early evidence of SHARE’s feasibility, acceptability, and effectiveness are promising (BLINDED) (Karlsson, Bridges, Bell, & Petretic, 2014; Karlsson, Zielinski, & Bridges, 2015, 2019), and SHARE has been sustained in the facility for near 8 years, leaving the program uniquely-positioned to inform knowledge on successful implementation and sustainment of specialty trauma therapies in CJ settings. Therefore, a member of the broader SHARE team initiated a research study to specifically examine the implementation and sustainment of SHARE (Zielinski et al., In press). The goal of the study was to gather information that could be used to inform implementation of SHARE in other CJ settings.

#### Examination of an implementation science research study in a CJ setting

Interviews were conducted to systematically assess and document the process by which SHARE was implemented and sustained at a women’s community correction center and to identify factors that influenced the implementation process. Because the aim of these interviews was to describe the process of implementing SHARE over time, including identification of strategies that were attempted or used to promote implementation of SHARE, the research team selected a *process framework,* EPIS (Aarons, Hurlburt, & Mc Cue Horwitz, 2011) to guide the study. The interview protocol was organized by the four phases of the EPIS model (i.e., *Exploration, Preparation, Implementation,* and *Sustainment)* and questions asked about factors associated with the outer system context, the inner organizational context, and the dynamic across contexts. Interview participants were 22 key informants and stakeholders, including individuals from the university, members of the treatment team, incarcerated women who may or may not have participated in the group, and community corrections center staff, leadership, and volunteers. Stakeholders (*n* = 13) were invited to complete an interview due to their role in SHARE implementation; some stakeholders were involved during all EPIS phases and others were selected because of their key role during specific phases. This approach to recruitment ensured that there were multiple people involved in each EPIS phase who could report the factors relevant to implementation, and that stakeholders only involved in one phase who had critical roles were not omitted. Incarcerated women (*n* = 9), some of whom had and had not completed SHARE, were also interviewed to understand the reasons that led them to enroll or not enroll in SHARE, and how SHARE was viewed by the intended recipients.

The interviews were first analyzed to identify: 1) factors that influenced implementation of SHARE at each EPIS phase and 2) strategies that were used to support SHARE implementation and sustainment.^1^ Incarcerated women provided valuable insight into the importance of correctional centers offering treatment for sexual violence victimization and the influential role of staff in raising awareness about programming options in helping them select programs in which to participate. Analyses also revealed the critical role of correctional-academic partnerships in developing and implementing SHARE at the community corrections center. Many carceral settings have active volunteer programs; this case study suggests that partnering with community-facing agencies and/or service-oriented graduate programs may be a way to sustainably expand intervention capacity in these under-resourced settings. This is especially true when the needs and resources of the partnering organizations are synergistic, as was found to be the case in SHARE implementation. Examination of inner context factors for each organization involved in developing and implementing THERAPY X revealed that implementation facilitators at times resulted from an interaction between more than one organization. For example, the clinically-trained leadership and rehabilitation focus of the community corrections center were seen as facilitators to SHARE implementation in the early days (*Exploration* and *Preparation* phases), though it was acknowledged that it was still a prison and it was critical for center leadership to problem-solve and make some accommodations to facilitate SHARE (*Implementation* phase). Other implementation facilitators were specific to a single organization. For example, one critical facilitator in SHARE implementation was that the local university clinical psychology program provided a steady stream of students with both the need for clinical hours/training and the desire to lead a psychotherapy group for incarcerated survivors of sexual violence (*Sustainment* phase).

Overall, we found that the SHARE implementers used at least fifteen discrete implementation strategies throughout different EPIS phases. The strategies used included identifying and preparing champions and opinion leaders in the community corrections center during the *Exploration*, *Preparation*, and *Implementation* phases, obtaining and using participant feedback to improve SHARE materials and the enrollment process during the *Implementation* phase, and purposively reexamining and tailoring strategies throughout the *Implementation* and *Sustainment* phases. Use of many strategies is common in successful implementation efforts (c.f. Rogal et al., 2017), something that CJ settings wishing to implement new programs must consider.

The full results of the study described in this case example are available at BLINDED in Zielinski et al. (In press); here, we have provided a brief illustration of how purposely examining the implementation of successful programs—guided by an implementation science framework—can lead to important knowledge of what it takes to sustain programs within local CJ settings. Because the case study was retrospective and limited to one site, we did not use surveys to measure factors such as organizational climate/culture. Such surveys are available and may be a valuable tool when applied earlier in implementation or during multi-site implementation efforts. Our findings may yet be useful to other CJ settings with similar populations, organizational characteristics, or community resources. Notably, the results of implementation studies can also inform practical products to aid in future implementation efforts. For example, the results of the investigation we described here are currently being used to create an implementation toolkit to accompany the SHARE manual to aid other CJ settings in their implementation of this therapy group.

## Discussion and conclusions

These case examples offer insight into how implementation frameworks can be used to examine implementation successes and failures within CJ settings. In case example 1, the domains of CFIR could have been used to help guide the county jail in investigating what was causing low screening rates and identify ways to overcome these issues moving forward. Even better, CFIR could have been used in advance of implementing mental health screening in the jail to understand the barriers unique to that setting so that strategies could have been planned beforehand. In case example 2, a retrospective process evaluation was conducted to better understand how and why a trauma therapy has been successfully sustained in a community corrections center over time so that other facilities might learn from their success. Together, these case examples demonstrate the importance of using IS frameworks in identifying the factors influencing uptake or sustained use of mental health innovations in unique CJ contexts, as well as the strategies that enhance implementation and overcome obstacles that arise. This information can be used to plan implementation efforts, improve existing implementation efforts, or inform future implementation in similar CJ settings.

The challenges that CJ systems face when attempting to integrate evidence-based and innovative practices are substantial. These challenges demand that we incorporate knowledge from implementation science researchers in other fields. The implementation influences (i.e., barriers and facilitators) found to be important in our case examples may be directly applicable in similar settings and implementation climates. Regardless, these case examples illustrate both the value of bringing implementation science to criminal justice work and examples of how this can be done in varying levels of depth—an important prospect given that evidence-based practices to address behavioral health conditions are often absent or inconsistently applied in CJ settings. Even easily remedied factors, such as a lack of staff training to administer a brief screening tool, can have a major impact on whether important health improvement initiatives ever make it into practice or are sustained. By applying implementation science frameworks and assessing barriers *before* implementation rather than retrospectively, researchers can help accelerate translation of evidence-based practices into standard of care. However, given that applications of implementation science to CJ settings is nascent, the time is ripe for sharing knowledge gained from implementation successes—and even failures—in this unique context. Strategies that have been well-defined and studied by implementation science may provide direction for *how* to approach integration of important innovations within the context of barriers that may not be possible to fully remedy. Moreover, as applications of implementation science to CJ settings grows, we may uncover strategies that are unique to or especially pertinent to atypical contexts for health care like prisons and jails.

Importantly, the highly collaborative nature of implementation science research and practice will increase the likelihood that stakeholders will meet the needs of the specific context. This is particularly true for the context of CJ settings where strong collaborations are of critical importance. Given that the need to identify and address systemic barriers to evidence-based practices is not unique to CJ settings, such settings can clearly benefit from the advances the field of implementation science has made with respect to developing the strategies, tools, and processes that allow systems to increase uptake of much-needed interventions. However, CJ settings do face unique barriers that are underrepresented in the extant implementation science literature (e.g., emphasis on sanctions and control, limitations on intervention materials in carceral environments). Research that articulates the application of implementation science in CJ settings is sorely needed if we are going to break the cycle of repeated incarceration for individuals in this extraordinarily vulnerable population.

In closing it is worth noting that this paper is in no way a comprehensive review of all that implementation science has to offer if more routinely applied to or studied in CJ settings. There are a host of introductory readings on implementation science theories, models, and frameworks; for a basic introduction, novice readers may find Nilsen (2015) and Damschroder (2020) reviews a to be helpful resources regarding how to select a type of theory that is relevant to the implementation question or effort at hand. For a compendium of possible implementation strategies, we recommend Powell et al. (2015) manuscript. Our paper has primarily highlighted the use of qualitative methods in implementation work; however, there are also many survey and observational measures that can be used to quantitatively assess factors such as the perceived acceptability, appropriateness, and feasibility of adopting an evidence-based practice as well as organizational-level factors relevant to implementation (e.g., leadership engagement, organizational culture/climate). Such measures are important given the influential role of organizational context in determining the successful implementation and sustainment of evidence-based practices (Aarons et al., 2011; Glisson, 2007). Measure repositories, many of which can be found freely available on the web, may be particularly helpful for those who are new to such measures.^2^

To the authors knowledge, there is not yet a systematic review of the use and effectiveness of specific implementation strategies, theories, and outcomes within CJ settings. Such a review would be a valuable addition to the literature, especially given overlaps between implementation science and process improvement strategies—the latter of which may have been used more routinely in CJ settings previously. Our hope is that this paper will inspire CJ researchers to routinely include implementation research questions in to their work. We also hope that they will share the knowledge they gain with CJ leadership and staff to support change within a system that is beginning to recognize its role in treatment rather than punishment alone.

## Data Availability

Not applicable.

## Footnotes

1 To accomplish this, the researchers designated segments of the data as relevant to EPIS phases *(Exploration/Preparation*, *Implementation*, and *Sustainment*) and contexts (Inner Setting, Outer Setting) for each of the three partnering organizations, as well as identified implementation strategies discussed. Then, all contextual segments were re-reviewed and inductively coded for factors (e.g. implementation facilitators and barriers) related to these EPIS constructs with descriptive summary and sub-codes. The codebook was established in an iterative process of discussion and refinement.

2 See https://www.c4tbh.org/resources/measures-for-implementation-studies/ and https://episframework.com/measures as example compendiums.
